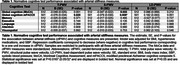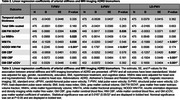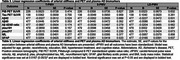# Relationships of structural and load‐dependent arterial stiffness with cognition and AD/ADRD biomarkers: The Healthy Brain Study

**DOI:** 10.1002/alz70860_101143

**Published:** 2025-12-23

**Authors:** Theodore M DeConne, Cynthia Suerken, Megan E. Lipford, Sam N. Lockhart, Trey R. Bateman, Mark A. Espeland, Marc D. Rudolph, Courtney L. Sutphen, Thomas C. Register, Michelle M Mielke, Ryan Pewowaruk, Adam D Gepner, Laura D Baker, Suzanne Craft, Timothy M. Hughes

**Affiliations:** ^1^ Wake Forest University School of Medicine, Winston‐Salem, NC, USA; ^2^ Wake Forest School of Medicine, Winston‐Salem, NC, USA; ^3^ Division of Public Health Sciences, Wake Forest University, School of Medicine, Winston‐Salem, NC, USA; ^4^ Ryan Pewowaruk Research Consulting, Madison, WI, USA; ^5^ University of Wisconsin School of Medicine and Public Health, Madison, WI, USA; ^6^ Wake Forest University, Winston‐Salem, NC, USA

## Abstract

**Background:**

Recent advancements allow the delineation of load‐dependent (LD‐) and structural (S‐) arterial stiffness from total pulse‐wave velocity (T‐PWV), providing a greater understanding of how arterial stiffness augments the risk for AD/ADRD. Based on prior data, we tested the hypothesis that higher S‐PWV, but not LD‐PWV, would be associated with lower cognitive function, and worsened MRI imaging biomarkers and plasma and PET AD biomarkers.

**Method:**

Data from 516 participants enrolled in the Wake Forest Alzheimer's Disease Research Center's Healthy Brain Study were included in this analysis (70.4±8.1 years old, 67% women, 287 cognitively normal, 229 cognitively impaired or demented). Arterial stiffness was measured using carotid‐femoral PWV. S‐PWV and LD‐PWV were calculated using participant‐specific exponential models. Global cognitive function was measured using the MoCA and mPACC5, and cognitive domain scores were calculated from the Uniform Data Set version‐3. 3T Brain MRI outcomes were derived from T1, T2 FLAIR, DTI/NODDI, and ASL. Whole brain Aβ‐PET and Tau‐PET were measured using [11C]‐Pittsburgh compound‐B) and [18F] Flortaucipir, respectively. Multivariable linear regression models were used to relate arterial stiffness to all outcomes adjusting for relevant co‐variates (Tables 1‐3). Plasma AD biomarkers (Aβ40, Aβ42, NFL, GFAP, ptau181, and ptau217) were measured using Quanterix SIMOA assays.

**Result:**

We observed that higher T‐PWV and S‐PWV were associated with lower global cognition function (Table 1). Higher LD‐PWV was associated with lower white matter fractional anisotropy and gray matter and white matter cerebral blood flow, while higher T‐PWV and S‐PWV was associated with higher gray matter free water. Higher T‐PWV was also associated with lower white matter fractional anisotropy and higher white matter free water and hyperintensity volume (Table 2). Lastly, we did not observe any significant associations between arterial stiffness and plasma or PET AD biomarkers (Table 3).

**Conclusion:**

Higher T‐PWV, primarily due to structural remodeling, was associated with lower cognitive scores and higher white matter and gray matter structural abnormalities. In contrast, stiffness associated with blood pressure was associated with abnormalities in white matter integrity and abnormalities in white and gray matter blood flow. Interventions targeting arterial stiffness may be warranted to preserve cognition and cerebrovascular health.